# Input Subsidies to Improve Smallholder Maize Productivity in Malawi: Toward an African Green Revolution

**DOI:** 10.1371/journal.pbio.1000023

**Published:** 2009-01-27

**Authors:** Glenn Denning, Patrick Kabambe, Pedro Sanchez, Alia Malik, Rafael Flor, Rebbie Harawa, Phelire Nkhoma, Colleen Zamba, Clement Banda, Chrispin Magombo, Michael Keating, Justine Wangila, Jeffrey Sachs

## Abstract

Recent hikes in food prices have created economic and social turmoil in many African countries. But in Malawi, fertilizer and seed subsidies have enabled small-scale farmers to improve maize productivity and achieve food security.

Emerging from the worst harvest in a decade, the Government of Malawi implemented one of the most ambitious and successful assaults on hunger in the history of the African continent. Through a national input subsidy program, coinciding with better rainfall conditions, maize production doubled in 2006 and almost tripled in 2007. From a 43% national food deficit in 2005, Malawi achieved a 53% surplus in 2007, some of which was exported to neighboring countries. An associated decline in the price of maize conveys important benefits to low-income urban and rural households that are net food consumers. Malawi's recent experience may provide important lessons for achieving food security through smallholders in Africa.

Agricultural productivity improvements have long been viewed as the foundation for economic prosperity and social development [1–3]. Asia's Green Revolution began in the 1960s with the development of fertilizer-responsive, high-yielding varieties of rice and wheat [4]. Global average yields of these staple crops more than doubled over this period with greatest impact in regions with irrigation or more reliable rainfall. Improved access to fertilizer through state-supported subsidies, rural credit, and improved infrastructure contributed to strong productivity growth in both crops. Asian governments also supported the uptake of new technology through research and extension, and intervened in the market though price support [5,6].

In contrast, agricultural productivity growth in sub-Saharan Africa has not kept pace with population growth. The per capita growth rate of agricultural gross domestic product was negative during the 1980s and 1990s, though improvements have been noted since 2000 [6]. Production growth of the major African food crops (maize and root crops) was based almost entirely on extending the cultivated area, with only minor contributions from yield growth [4]. Poor infrastructure and related high transport costs (for both inputs and surplus production), inadequate institutional support (credit and extension), political instability, diverse agroecological complexities, low fertilizer use, and the limited availability of suitable high-yielding varieties have all contributed to low agricultural productivity growth in Africa [4–6].

The slower productivity growth in Africa compared with Asia masks a number of limited successes that could point to a latent African Green Revolution. Recognizing the role of agriculture in stimulating economic growth and reducing rural poverty, African governments promoted fertilizer use during the 1970s and early 1980s [7] through several interventions, including direct subsidies that reduced fertilizer prices for farmers, government-financed and -managed input credit programs, centralized fertilizer procurement and distribution, and control of output markets.

Impressive improvements in maize productivity were demonstrated in Kenya, Zimbabwe, and Zambia during the 1980s [8]. Cereal crop output in Ethiopia has dramatically increased over the past decade [9]. Several other studies have shown the potential of input subsidies in accelerating crop production [10–12]. However, these positive results were generally not sustained with the advent of donor-driven structural adjustment and the dismantling of government-supported institutions and subsidies.

By the turn of the century, fertilizer use in Africa was only 8 kg/ha, compared with 96 kg/ha in East and Southeast Asia and 101 kg/ha in South Asia [7]. Today, Africa accounts for less than 1% of global fertilizer consumption. A World Bank synthesis of lessons learned from earlier efforts to promote fertilizer use on the continent [7] attributed this failure to high and unsustainable fiscal and administrative costs, governments' weak capacity to implement programs, and governments' inability to take account of the diversity of production systems and farmers' needs.

Donors, led by the World Bank, argued for the abolition of state-led interventions including subsidies. As a result, many government input supply agencies were dissolved or privatized. Under these circumstances, fertilizer costs rose sharply and constrained adoption of fertilizer use by small-scale farmers. The World Bank study concluded that “although these reforms had generated positive impacts on government budgets, they resulted in significant reductions in overall levels of fertilizer use and increased food insecurity among many rural households” [7, p. 4]. This policy failure caused a serious reassessment among governments, creating the setting for a return to subsidies as a potential intervention for promoting food security and agricultural growth.

Against the broader continent-wide trend, fertilizer use by smallholder farmers in Kenya has increased dramatically since the early 1990s [7]. This apparent anomaly has been attributed to four main factors: stable fertilizer market policies leading to rapid expansion of private fertilizer distribution networks; reduction in the average distance between farmers and fertilizer retailers; greater competition among importers and wholesalers; and high profitability of Kenya's horticulture industry leading to maize-horticulture intercrops [7]. However, this country-level impact of market liberalization remains concentrated in more favorable and wealthier regions of the country where farmers earn sufficient cash from other enterprises, such as horticulture and dairy farming, to buy maize fertilizer. In one of Kenya's poorest and most food-insecure regions, the western lowlands, 87% of smallholders use no fertilizer on maize [13]. While generally praising Kenya's progress in fertilizer market liberalization, a recent study of fertilizer policies and use in Kenya [13] acknowledged the fragility of the Kenyan success story and concluded: “Because mean household incomes are higher in Kenya compared with many other African countries, the impressive market-led growth in smallholder fertilizer use in Kenya may not be easily transferable to areas where effective demand is highly constrained” [13, p. 39].

In Malawi, recent success with input subsidies highlights how pro-poor public investments in maize productivity improvement can be made cost-effectively. This essay examines the circumstances, results, and implications from the 2005–2006 and 2006–2007 national input subsidy programs, and describes the experience of more intensive support undertaken by the Millennium Villages Project (MVP) in one district of southern Malawi.

## Smallholder Maize Production in Malawi

Malawi is a landlocked tropical country of more than 13 million people. Its economy is heavily dependent on agriculture, which employs 78% of the national labor force [14]. Maize is grown by 97% of farming households and accounts for 60% of total calorie consumption. Almost all maize is grown without irrigation during the single rainy season from October to April, which is subject to rainfall variability that can be particularly damaging when short dry spells occur during the critical flowering and early grain filling stages [15].

Decades of intensive cultivation by smallholders, in the absence of significant fertilizer use, have depleted soils of nutrients, particularly nitrogen [16,17]. National yields of maize have averaged 1.3 metric tons per hectare (t/ha) during the last 20 years [9]. In contrast, the average yield of rainfed maize in Iowa in the United States (1997–2007) exceeded 10 t/ha [18]. Over half of Malawi's farming households operate below subsistence. Because of low productivity and small farm size, only 20% of maize farmers produce surplus and sell their product. On-farm storage losses are high. As a result, most households purchase maize at much higher prices when stocks are exhausted, typically during January to March [14].

To cope with food deficits, households reduce daily maize consumption, increase consumption of alternative calorie sources (such as cassava), sell assets (such as livestock), and seek employment on large commercial estates or in towns [14]. Theft of crops in the field is common during severe food shortages, prompting farmers to harvest unripe green maize for immediate consumption. Food insecurity also encourages unsafe sexual practices leading to higher incidence of HIV/AIDS, other sexually transmitted diseases, teenage pregnancies, and abortions [19]. Gender and theft-related violence increase. And school attendance usually drops during times of food shortage.

These dire circumstances underscore the urgent need to improve smallholder maize productivity through an African Green Revolution [20]. The technology and the knowledge to improve maize yields in Malawi have existed for at least three decades [21,22]. The challenge, identified in July 2004 by then United Nations Secretary-General Kofi Annan, is to “turn this knowledge into practice” and thereby “take the first steps out of chronic poverty” [20, p. 19].

## 2005 Food Emergency Leads to a Bold Policy Decision

**Figure 1:**
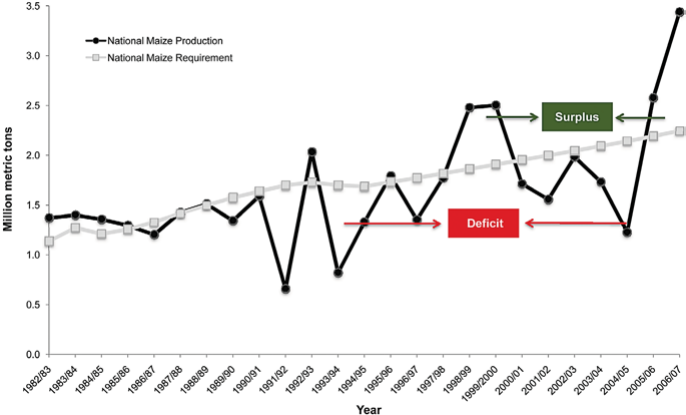
National Maize Production and Maize Food Requirement over 25 Years Compiled from [9,49]. Unusual dips and spikes in maize requirement are a reflection of inconsistency in methods of national population determination.

The 2004–2005 maize season (planted October–December 2004 and harvested April–June 2005) was the worst in a decade (Figure 1). Many parts of the country went without rain for up to one month during January and February—the critical tasseling and ear development stages for maize—with a devastating effect on yields: the national average was only 0.76 t/ha, 40% below the long-term average. Total maize production for 2004–2005 was 24% less than the previous year, amounting to 57% of the estimated national maize food requirement [9].

In May 2005, the Malawi Vulnerability Assessment Committee concluded that over 4.2 million people required food aid [23]. The food situation was deteriorating rapidly, and a major humanitarian relief operation began [24]. By November 2005, as the maize prices in local markets continued to rise, the estimate went up to 5 million Malawians—38% of the population—in need of food aid [15].

In response to recurring food deficits, the Government decided to invest in subsidizing agricultural inputs, an approach that was being vigorously promoted by the UN Millennium Project [25]. This policy attracted objections from some major donors [26] who were concerned about the potential cost and the absence of a clear exit strategy. Those same donors had earlier supported a Starter Pack program in 1998–1999 and 1999–2000 whereby small packages of fertilizer, maize seed, and legume seed, sufficient for 0.1 ha, were distributed free to almost all maize smallholders in Malawi [27]. This program led to an extra 280,000 to 420,000 t maize produced. However, the main donors scaled down their support to Starter Pack, citing operational weaknesses, lack of targeting to the poorest households, and the negative impact on diversification efforts [28]. The sharp reduction in the coverage of the program was reflected in national production statistics (Figure 1). Malawi once again fell below self-sufficiency and resumed its dependency on food aid [19,27].

In the face of adverse donor reactions, and after heated parliamentary debate, the Government used discretionary budget funds and support from the UN to import fertilizer and procure improved maize seed for distribution to farmers. Through the national input subsidy program, the Government allocated coupons to buy sufficient fertilizer to grow maize on one acre (0.4 ha), a 4-fold increase in the amount provided under Starter Pack, as well as 3 kg of maize seed—an insufficient amount (10 kg of seed are needed for 0.4 ha) necessitated by funding constraints. Seed, of a limited number of Government-recommended open pollinated varieties (OPVs), was sold at less than one third of the market rate. The total market value of the inputs was MK5,500 (US$44.00), of which the farmers paid MK2,050 (US$16.40), representing an overall 63% subsidy. Coupons were allocated across regions and then distributed to districts and traditional authorities (sub-district government entities), who allocated them to Village Development Committees, which identified the recipients. All of the subsidized fertilizer and seed was distributed through government agencies [29].

Including late distribution of supplementary coupons, 3.4 million coupons were issued (73% of these were for maize cultivation), of which 75% were redeemed [29]. A total of 132,000 t fertilizer (22,000 t of which were for tobacco fertilizers) and 6,000 t of improved maize seed were made available. The total cost of the program was estimated at MK7.2 billion (US$58 million), representing the direct costs of purchase and distribution of fertilizer, net of sales receipts [29]. Excluding the cost of tobacco fertilizer, the cost of the maize subsidy in 2005–2006 is estimated at approximately US$50 million.

There was no explicit targeting of subsidies toward the poor, who represented 54% of the population in 2005 [14]. This was another criticism from donors and a reason for their slow support. However, there was officially a maximum allocation of two 50 kg bags per household. This upper limit was intended to reduce the potential for capture of subsidies by larger farmers.

## A Bumper Harvest in 2006

The 2005–2006 season had good rains, and total maize production was more than double the 2004–2005 harvest, producing a surplus of 510,000 t above the national maize requirement (Table 1). Maize yields averaged 1.59 t/ha, doubling the 0.76 t/ha of the drought-affected 2004–2005 season. Incremental maize production attributed to the fertilizer subsidy was estimated at 300,000 to 400,000 t [30].

**Table 1:**
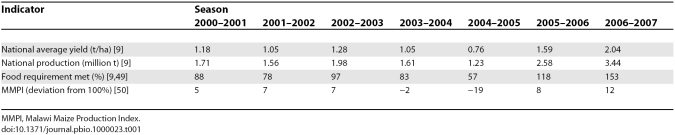
Malawi's National Maize Production, Food Self-Sufficiency, and the Malawi Maize Production Index, 2000–2001 to 2006–2007

The 2005–2006 growing season was clearly more favorable for maize production than the drought-affected 2004–2005 season but was broadly comparable to the 2001–2002 and 2002–2003 seasons, when relatively small subsidies were provided. Maize production in 2005–2006 was higher than in the 2001–2002 and 2002–2003 seasons by 1.02 million t and 600,000 t, respectively, suggesting a large incremental impact of the subsidy beyond the effect of better rainfall.

Encouraged by this achievement, the Government continued the subsidy program for the 2006–2007 season. While donors acknowledged the impact of the 2005–2006 subsidy, they argued for increasing the participation of the private sector, especially the agro-dealer network. The Government recognized the need to partner with the private sector, but expressed concerns in the event of unsold government stocks, unreliable seed quality, and inability to reach the more remote areas that were not served by the private sector. After the UK Department for International Development offered technical and financial support, a compromise was reached whereby the Government was financially buffered against possible unsold stocks, input distribution was supported by a professional logistics unit, and seed and fertilizer quality was monitored.

## Continued Success in 2007

For the 2006–2007 season, the input subsidy program was repeated on a similar scale to 2005–2006. A total of 3.5 million coupons for maize fertilizer were distributed, targeted to maize-growing households, to enable purchase of the same amounts of fertilizer at the same price as in the previous year. Two million seed coupons were also distributed, each enabling recipients to obtain 3 kg of OPV seed or 2 kg hybrid seed, depending on the farmers' choice and seed availability. According to Government of Malawi estimates [29], overall coupon redemption was 95% for fertilizer and 91% for seed, higher than in the previous season. The Government met 87% of the net subsidy cost. The private sector distributed 28% of the fertilizer and all of the seed. Seventy-six percent of farmers opted for the higher-yielding hybrids over the less expensive OPVs [29], challenging perceptions among some donors and nongovernmental organizations that hybrid varieties were inappropriate for small-scale farmers (Box 1).

**Box 1: OPVs versus Hybrids**

In Malawi and throughout Africa, there is debate about the relative merits of OPVs and hybrid varieties of maize [45]. Smallholder maize production in Africa has traditionally been based on the use of OPVs, whereby the seed is retained from year to year. Over time, through farmer selection, these traditional OPVs, known as landraces, become well adapted to the particular farm environment. Improved OPVs have been bred and selected for special characteristics such as drought tolerance and disease resistance. Seed can be recycled by farmers for a maximum of three years without significant yield loss. OPVs typically yield 10%–25% less than hybrids [45].

Hybrid maize is produced by crossing two genetically unrelated inbred parents. The resulting seed exhibits hybrid vigor, but recycled hybrid seed will not breed true in subsequent generations, and can result in yield losses of 30% or more, reducing and perhaps eliminating any yield advantage in subsequent planting [45]. Hybrids are more uniform and generally higher yielding than OPVs. The Government of Malawi launched a hybrid breeding program in 1950, following a severe drought in 1949 [46]. The first hybrids were released in 1958 and by the 1990s were adopted by about a quarter of Malawi's smallholders. Research indicated a consistent yield advantage of hybrids over local maize varieties at all levels of fertilizer use, including in a drought year [22,47]. This yield advantage of hybrids remained even under low soil fertility and drought conditions.

On balance, the yield advantage of hybrids appears robust for smallholder maize production in Malawi. However, the choice of hybrids versus OPVs is constrained by a complex set of factors including the higher seed cost and the often poorer storage and processing characteristics of hybrids [47]. During the second year of the Malawi input subsidy program, voucher recipients were given a choice of OPV or hybrids: 2 kg of hybrid seed or 3 kg improved OPV, depending on supplier costs. Based on coupon redemption, 76% of farmers chose hybrids over OPVs [29]. Thus, in Malawi, there is clearly a strong farmer preference for hybrids over improved OPVs provided that the prices of seed and fertilizer are subsidized.

Hybrid seed is generally more expensive than OPVs because of the higher cost of seed production and private sector control over supplies. Farmers in lower potential environments often find it difficult to recover the costs of hybrid seed and fertilizer. In the absence of deep subsidies to both seed and fertilizer, risk perceptions of small-scale farmers, especially in low potential rainfed environments, have been shown to constrain adoption of hybrid maize [47,48].

The 2006–2007 harvest was estimated at 3.44 million t, an all-time national record for Malawi, generating a surplus of about 1.34 million t of maize grain above national requirements (Table 1, Figure 1). The incremental effect of the fertilizer subsidy on maize production was estimated at 670,000 t for 2006–2007, valued at US$117 million in additional crop production, assuming a maize producer price of US$175/t. The total program cost in 2006–2007 was US$72 million [29], approximately US$62 million of which was directed to maize fertilizer and seed costs. By late 2007, Malawi had exported over 300,000 t of maize to Zimbabwe, not only generating income for its smallholder farmers, but contributing to regional food security [15]. The Government decided to continue the program in 2007–2008.

The poor harvest of 2004–2005 led to a sharp rise in the price of maize sold at local markets throughout Malawi [15]. By June 2006, with most of the 2005–2006 crop harvested, the average price had dropped by 61%. With the effects of the 2006–2007 bumper crop, the maize price dropped still further. Anecdotal evidence pointed to a modest increase in maize prices in some markets during August–October 2007 due to procurement for export to Zimbabwe. Since then, global food shortages have maintained prices at levels attractive to farmers [15].

These results suggest that the maize consumers in Malawi have benefited from the two successive strong harvests and the related price declines. This outcome is fully consistent with experience in Asia [4] and suggests an important potential impact of seed and fertilizer subsidies on food security for the poorest households that are net consumers even after good harvests. Further research is needed to understand the impact of the subsidy on maize consumers.

Displacement of commercial sales by subsidized fertilizer was estimated at 60% in 2005–2006 and 54% in 2006–2007 [29]. While this raised concerns from the business community, the results must be weighed against the net social impact of achieving national food security. Experience from the MVP (below), which operates in low-income “hunger hot-spots,” is revealing that commercial sales actually increase in the poorest areas that were previously unserved or underserved by agricultural input dealers. In time, stimulating agricultural productivity will likely increase commercial activity in rural areas and extend new opportunities for agricultural input suppliers.

## Millennium Villages: Deepening and Broadening Rural Investment

Concurrent with the implementation of the national input subsidy program, the MVP was established in Malawi. The MVP is an integrated rural development approach that supports public-sector investments, leading to increased private-sector saving and investments [31]. The MVP began a year earlier in Sauri Village in western Kenya, achieving average maize yields of over 5 t/ha, and meeting 166% of basic caloric requirements [31]. Inspired by Sauri's early success, the MVP employed a similar approach in Malawi. A total of 11,000 farming households in and around the village of Mwandama in Zomba District were identified for a program of intensified multisectoral support beginning in the 2005–2006 season.

During August to October 2005, several rounds of consultation were held with the Mwandama community. These revealed major concerns about food security and the ability to recover from the 2004–2005 crop failure. Women were especially vocal about food shortages. Despite their desperation, farmers expressed an urgent need for seed and fertilizer over food aid, although aid was also required for the worst affected households. However, most farmers in these severely affected areas did not even have enough money to take advantage of the national fertilizer subsidy, revealing the need to modify the policies of the national input subsidy program.

Instead of purchasing inputs at subsidized rates, each household in the Millennium Villages received 10 kg of hybrid maize seed and the recommended fertilizer inputs for a typical smallholder farm of 0.4 ha. Farmers were also trained in the “Sasakawa” planting method advocated by Sasakawa Global 2000 [32], using closer ridge spacing (75 cm apart) and single seeds (25 cm apart) instead of the traditional method of planting multiple seeds 50 cm apart along the ridges. The main differences with the broader national subsidy program were (1) enough seed was provided to plant 0.4 ha maize, and (2) farmers were not required to pay for the fertilizer up front. Instead, after harvest, the recipients were required to repay a portion of the input cost (around 30%) in kind to a school meal program to be implemented in their villages.

Farmers welcomed the availability of seed, fertilizer, and extension services. Aided by a better than average rainy season, the intervention package resulted in unprecedented productivity improvements. In Mwandama Village, 1,000 farmers obtained an average yield of 6.50 t/ha—more than four times the officially estimated national average for 2005–2006. In the broader cluster of 11 Millennium Villages around and including Mwandama, 11,000 farmers averaged a yield of 5.18 t/ha (random sample of 90 fields), compared with a yield of 2.21 t/ha (random sample of 30 fields) from non-intervention areas (where farmers had access to the national subsidy program) (Table 2).

**Table 2:**
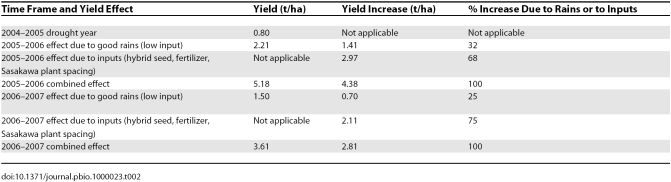
Partitioning the Yield Increases at Mwandama Cluster of 11,000 Farms

The interventions were repeated for the 2006–2007 season. The only difference was the addition of 3 kg groundnut seed and 2 kg pigeon pea seed to encourage crop diversification. The yields obtained in 2006–2007 dropped sharply to 3.61 t/ha, primarily because of poor seed germination due to a two-week dry spell following the germinating rains. However, even under these circumstances, farmers in the Mwandama cluster gained 2.11 t/ha from the additional inputs (Table 2).

To estimate the relative effects of rainfall and inputs (here defined as the combination of hybrid seed, fertilizer, and Sasakawa plant spacing) on maize yields, the 2004–2005 drought-affected crop that received little fertilizer was used as a baseline. The rainfall effect for the 2005–2006 season was then estimated by comparing the 2004–2005 yield (farmers' estimates) with the 2005–2006 estimates based on crop cuts of fields adjacent to the Mwandama MVP cluster. Using this simple estimation technique, the rainfall effect is 32%. The balance of 68% accounts for the use of improved inputs. The results for 2006–2007 indicated a similar effect. Thus, in both years, the rainfall effect appears to account for 25%–32% of the yield increase. The balance can be attributed to the use of improved seed, fertilizer application, and related extension advice.

The impact of the interventions in the MVP undoubtedly means the difference between a food deficit experienced in “normal years” and a significant surplus above normal consumption needs, which is about 1,000 kg/year for the average family farm size of 5.5 people. Older farmers reported that they had never before experienced or even seen maize crops like these, except on nearby large commercial estates.

The inclusion of the Millennium Villages data complements the experience gained from Malawi's national input subsidy program. The results demonstrated that there remains a very large yield gap between maize yields achieved at the national level (even with a substantial subsidy) and potential yields based on whole farm intensified support.

## Lessons Learned

### Political will and action provide the foundation for change.

The use of fertilizer subsidies had been actively discouraged by donors for two decades. The perceived high cost and trade-offs with other development investments, the absence of clear time-bound exit strategies, and difficulty targeting the poorest of the poor have led many donors to withhold their support to input subsidy programs. Anti-subsidy bias was well established in Malawi in 2005, and there was little encouragement from donors for the Government to implement the program described in this essay. At the Africa Summit of the World Economic Forum in Cape Town, South Africa, referring to the situation in Malawi in 2005 and his commitment to act and provide input subsidies, President Bingu wa Mutharika said: “Enough is enough. I am not going to go on my knees to beg for food. Let us grow the food ourselves. And indeed we have.” [33].

This political leadership has effectively buffered Malawi from the economically and socially destructive effects of the global food price increases of 2007 and 2008. Carryover stocks have meant that Malawi is no longer dependent on food aid and costly commercial imports. Importantly, Malawi is now the focus of international attention as a result of some high-profile coverage in the international press [34,35]. Initially with some reluctance, but with increasing interest and engagement, several donors have begun to work with the Government to support and improve the effectiveness of the input subsidy program for smallholders.

### A national-scale program of this urgency is feasible although operational challenges remain.

Despite the enormous human resource capacity and infrastructure challenges facing Malawi, this national scale input subsidy program has been a success as measured by unprecedented production levels. With modest technical and financial support from donors, the logistical challenges of reaching over 2 million smallholder farmers were overcome during the Starter Pack program (1998–1999 and 1999–2000) [27] and again in 2005–2006 and 2006–2007 through the national input subsidy program. A number of operational challenges have been identified [29] and are being addressed by the Government in redesigning the program. These included: delays in program design and implementation leading to delayed delivery of inputs in some areas; cumbersome coupon processing and redemption systems; the need to improve program information sharing with the intended beneficiaries and general public; and shortages of fertilizers and mismatch of coupons and fertilizer types in some areas. Two major remaining challenges are the absence of private agro-dealers in remote rural areas and limited human and financial capacity of government agencies to meet the operational demands of the program [29]. However, each year of implementation has resulted in design improvements that are now inspiring other countries to emulate the Malawi experience.

### Knowledge exists to increase smallholder maize productivity and sharply reduce food insecurity.

The interventions implemented are straightforward practices that were developed by Malawian researchers and their international partners. The central biological basis for productivity improvement is the response of maize to nitrogen fertilizer application. The widespread occurrence of nitrogen deficiency in Africa and the availability of well adapted maize varieties, both hybrids and OPVs, mean that Malawi's experience is relevant beyond its borders. Importantly, these improved practices have been successfully applied by smallholders, suggesting that development of large-scale commercial farming may not be essential for the achievement of national or household food security in Africa. Indeed, questions may be raised as to whether large estates that currently occupy over 1 million ha in Malawi are an efficient use of the country's limited land resources.

### Supporting inputs rather than output subsidies, such as food aid, makes economic sense.

The results from the MVP showed that the provision of about US$60 of inputs (seed and fertilizer based on full unsubsidized cost) generated an extra 0.8 to 1.2 t of maize for a farmer on 0.4 ha. This extra food can be valued at US$140 to US$210 based on local prices during lean times (assuming US$175/t), a benefit to cost ratio of at least 2.3 in just six to nine months. While the external evaluation of the 2006–2007 subsidy program estimated a modest benefit/cost ratio of 0.76 to 1.36 [29], the indirect benefits on education, health, and social stability were not included. Moreover, this financial analysis did not consider the future stream of benefits flowing from the long-term rural economic transformation. The evaluation noted that the yield response to inputs, and thus the benefit to cost ratio, can be greatly improved by the timely delivery and use of inputs and better extension advice. The MVP demonstrated the impact of more efficient input use and therefore provides a standard against which improvements in national productivity can be measured.

### The cost of achieving food security is fiscally manageable and responsible.

The budgetary allocation, representing less than 7% of the 2005–2006 national budget (US$5/person/year), supplemented in 2006–2007 by donor support (less than US$1/person/year), is a remarkably small price to pay for achieving national food self-sufficiency and widespread household food security. By comparison, the cost of importing food in 2004–2005 was US$110 million (about US$8 per person). Donor aid to Malawi in 2005 was US$578 million or about US$44 per person [36]. On this basis, there should be little concern about the affordability of a program that has such profound and immediate impact on the lives of so many Malawians. Moreover, the export of surplus maize to Zimbabwe in 2007–2008 was estimated to generate over US$120 million. Nevertheless, the dramatic increases in fertilizer prices over the past 12 months require greater attention to fertilizer use efficiency and the use of complementary approaches to improving soil fertility (see later section on Future Challenges).

### Pro-poor “smart” input subsidies work.

Under the input subsidy program, most beneficiaries had access to the same quantity of inputs, irrespective of farm size. This implies that the smaller the farm, the greater the effective input level on a per hectare basis. During the past 20 years, large surpluses over requirements were obtained in 1993, 1999, 2000, 2006, and 2007, all years when large-scale input subsidies were implemented (Figure 1). The bulk of these surpluses was generated by smallholders. These results should not imply that fertilizer subsidies benefit all poor households. In cases where households have little or no land and/or are constrained by labor (e.g., as a result of sickness or household age structure), alternative approaches such as social cash transfer programs should be considered.

### Maize consumers are benefiting from lower prices, but future challenges will arise with continuing surplus production.

The direct production and livelihood benefits to most smallholder farming households are clear. However, the lower maize prices following the 2006 and 2007 harvests have likely improved food security for the urban poor and the vulnerable rural poor who remain net consuming households. This result mirrors experience from Asia's Green Revolution. Notwithstanding the recent spike in world food prices, continuing surpluses will demand greater attention to price stabilization mechanisms, improved post-harvest management, and incentives to diversify beyond maize to higher returning crops, including perennials and non-farm enterprises.

## Future Challenges: Sustaining a Green Revolution in Malawi

### Unreliable rainfall and climate change.

With almost all of its smallholder maize dependant on rainfall, Malawi is particularly vulnerable to large season-to-season variation in production. The returns on the fertilizer investment vary accordingly. The realization of Malawi's Green Revolution and its contribution to economic growth is also threatened by climate change. The Fourth Assessment Report of the Intergovernmental Panel on Climate Change highlighted the vulnerability of African agriculture and all who depend on it [37]. Agriculture in Malawi may be affected by shorter growing seasons and higher temperatures. The direct effects on agricultural production and food security will be exacerbated by greater exposure to malaria and other climate-influenced diseases that reduce labor productivity and employment opportunities.

Malawian farmers have no alternative but to adapt to climate change. Fortunately, several practical options for adaptation to Malawian conditions exist and need to be deployed as a matter of urgency [38]:

- Water harvesting, sustainable extraction of groundwater, conservation farming (reduced tillage, crop residue retention, and crop rotations), and improved water use efficiency in rainfed areas.
- Expanded irrigation through dams and extraction of water from Lake Malawi and the streams that feed it, subject to assessment of environmental impact.
- Shifts toward maize varieties with greater drought and heat tolerance, and improved pest and disease resistance, and corresponding adjustments in the national research agenda.
- Enterprise diversification toward higher value crops, value-adding, and off-farm employment that will generate income to buffer possible maize crop failures.
- Weather forecasting and provision of timely advice to farmers.
- Weather-related crop insurance.

A high priority of the Government of Malawi should be to design and implement a strategy to reduce rainfall-induced production variability and prepare farmers to adapt to climate change. This strategy will require major public investments on a scale comparable to those that have supported the national input subsidy program.

### High fertilizer prices.

Fertilizer prices rose dramatically in 2007 and 2008 [39], more than doubling the cost of the input subsidy program and leading to a projected budget shortfall of almost US$80 million. This rise in fertilizer prices highlights the vulnerability of Malawi to international market prices, a situation exacerbated by being landlocked. With high global food prices, the in-country production of maize by smallholders, even at three times the 2006–2007 cost of production, remains a more attractive and politically less risky proposition than international maize procurement and reliance on food aid.

While there is no immediate substitute for inorganic fertilizer, there may be scope to improve the efficiency of fertilizer use. Delayed access to seed and fertilizer is a recurring complaint of farmers in Malawi and elsewhere in Africa. The timing of fertilizer application can be improved as delayed application can sharply reduce uptake efficiency. This requires: early tendering, and contract signing by May each year; coupons to be distributed by early August; fertilizer and seed to be distributed by end of September; and stocks in the field to be replenished ahead of timely application.

The national input subsidy program should focus on the use of urea (46% nitrogen) because of its lower unit cost of nitrogen than the compound fertilizer known as 23-21-0 (which contains 23% nitrogen and 21% phosphorus). At mid-2008 prices, urea will cost 5% less and provide 33% more nitrogen than the current mix of urea and 23-21-0. As there are concerns about longer-term phosphorus deficiency in the absence of phosphorus application, this measure should be viewed as an interim solution only.

Inclusion of grain legumes in crop rotations can bring multiple benefits to smallholders. Groundnut, soybean, beans, and pigeon pea are well adapted to different parts of Malawi. These crops provide cash income and improved nutrition in addition to providing up to 60 kg nitrogen/ha of residual fertilizer equivalent to the soil [40]. A national crop legume promotion program is strongly recommended.

In the longer term, there is also scope to increase the contribution of nitrogen fixation through tree and shrub legumes. There is a long history of research on agroforestry in Malawi and neighboring countries in Southern Africa [41]. Availability of seed is often viewed as a constraint to adoption of agroforestry legumes in the region. One option for encouraging adoption is to subsidize agroforestry legume seed. Another is to exploit the untapped potential for supporting more efficient small-scale nurseries and seed producers [42].

### Post-harvest losses.

The surplus harvests in 2006 and 2007 have highlighted the need to improve post-harvest management of grain. The most common cause of post-harvest losses in Malawi is the larger grain borer (Prostephanus truncatus [Horn]), a storage pest that was introduced with food aid shipments [43]. There are no reliable national estimates of the losses caused by this pest. Without chemical treatment, household-level losses of 40% to 100% have been reported. Losses from other insect pests and rats are also widespread. Such losses erode much of the estimated net gain from the input subsidy program.

The Government recognized the importance and urgency of this problem by introducing post-harvest chemicals and distribution of improved storage bins in the 2008–2009 input subsidy program budget. However, a much greater effort is needed to educate farmers on effective and safe storage techniques and to ensure that the needed chemicals and storage facilities are made available.

## The Way Forward

After decades of food insecurity and recurring emergencies, the Government of Malawi has successfully implemented a national input subsidy program that led to major surpluses above national demand in 2006 and 2007. Drawing on their core resources—land and labor—and with a determination to be self-reliant and free of food aid, Malawian smallholders demonstrated that they have the ability to respond to strategic material support and incentives in order to contribute to their own well-being. Notwithstanding pockets of yield reduction and crop failure due to floods and drought, Malawi was blessed by two relatively favorable rainy seasons in 2005–2006 and 2006–2007. Some critics consider that Malawi was “lucky” in this respect. Yet, in rainfed agriculture, it is necessary to exploit better growing conditions when they arise.

Continuity of this highly cost-effective program is needed in order for Malawi to achieve its Green Revolution in a sustainable way. Solutions are still needed to address the risks of drought and dry spells, both through water management technologies and the spreading of financial risk. Improvements to fertilizer use efficiency and more targeted, locally refined recommendations need to be deployed. Agroforestry through the use of nitrogen-fixing trees is one of several options that can complement and ultimately reduce the need for inorganic nitrogen fertilizer. Post-harvest losses must be sharply reduced.

More generally, Malawi (and African countries like it) will require a firm 10- to 15-year multi-stakeholder commitment to support rural economic transformation from sub-subsistence farmers into diversified, small-scale entrepreneurs, including major complementary investments in health, nutrition, education, family planning, infrastructure, water, and sanitation. The input subsidy program must continue for several more years to meet the immediate needs for national and household food security, while longer-term investments in economic and social infrastructure are made. Any abrupt halt or downscaling of the subsidies, as was experienced by Malawi following two years of implementation of the Starter Pack program [19,27], would likely reverse the progress during the past three years and must be avoided. However, with food security stabilized, input subsidies can be gradually decreased and replaced by smallholder-focused rural credit that played an important role in Asia's Green Revolution. As in Asia, price support through strategic government procurement may also be required to stabilize prices during times of bumper harvests. The new Malawi Growth and Development Strategy [44] incorporates the fundamentals of this agriculture-led transformation and should be supported.

Malawi has led the way in Africa in demonstrating the opportunities and challenges of implementing a national input subsidy program. With the impetus of recent high food prices and a softening of donor opposition to subsidies, several of Malawi's neighbors (including Kenya, Rwanda, and Tanzania) are now studying, adapting, and building on this experience to design and implement similar programs for improving agricultural productivity. Malawi's experience will continue to provide valuable lessons for achieving and sustaining Africa's Green Revolution.

## Abbreviations

MVP: Millennium Villages Project
OPV: open pollinated variety
t/ha: tons per hectare
